# Microfluidic Leaching of Soil Minerals: Release of K^+^ from K Feldspar

**DOI:** 10.1371/journal.pone.0139979

**Published:** 2015-10-20

**Authors:** Davide Ciceri, Antoine Allanore

**Affiliations:** Department of Materials Science and Engineering, Massachusetts Institute of Technology, Cambridge, Massachusetts, United States of America; Texas A&M University, UNITED STATES

## Abstract

The rate of K^+^ leaching from soil minerals such as K-feldspar is believed to be too slow to provide agronomic benefit. Currently, theories and methods available to interpret kinetics of mineral processes in soil fail to consider its microfluidic nature. In this study, we measure the leaching rate of K^+^ ions from a K-feldspar-bearing rock (syenite) in a microfluidic environment, and demonstrate that at the spatial and temporal scales experienced by crop roots, K^+^ is available at a faster rate than that measured with conventional apparatuses. We present a device to investigate kinetics of mineral leaching at an unprecedented simultaneous resolution of space (~10^1^-10^2^ μm), time (~10^1^-10^2^ min) and fluid volume (~10^0^-10^1^ mL). Results obtained from such a device challenge the notion that silicate minerals cannot be used as alternative fertilizers for tropical soils.

## Introduction

Soil supports human development by providing society with essential benefits [1, 2]. Soil is a key subject of investigation with global issues such as climate change, food security and environmental degradation [3–8]. Leaching of soil minerals is a key step of weathering, a core soil process that transforms primary minerals (e.g., feldspars crystallized from magma) into secondary minerals (e.g., hydrated alumino silicates such as clays) through interactions with the soil solution. Understanding the dynamics of mineral leaching on both geological and human timescales is necessary to determine the ability of soils to fix toxic chemicals [1, 7, 8], to accommodate nuclear waste [9, 10], to sequestrate anthropogenic CO_2_ [3, 10–12] and to formulate affordable agromineral fertilizers [6, 13–15].

K-feldspar (KAlSi_3_O_8_) is a framework silicate that accounts for ~10 wt % of soils, constituting a key potassium reserve together with micas [16, 17]. Ground K-feldspar added to agricultural soils (stonemeal) has been discussed as a potential substitute for traditional potassium fertilizers, typically expensive soluble salts such as KCl, because of its abundance and relatively high K_2_O content (16.9 wt %) [13–15]. To date, a commonly cited limitation to such an approach is the slow leaching rate of K^+^ ions from the feldspar, determined by using either batch or flow-through apparatuses, which do not represent soil hydrodynamics [13, 14, 18]. A mass balance calculation shows that the amount of K-feldspar necessary to sustain the growth of a crop, such as leeks, scales with their leaching rate in soils (S1 Text; S1 Fig). Particularly, the rate at which K^+^ ions are available in the immediate vicinity of a root is decided in the microfluidic environment that surrounds it (S2 Text; S2 Fig). Packing of soil aggregates (250 μm‒2 μm) generates a network of pores (5,000 μm‒0.01 μm), which hosts the soil solution, and is the locus of both mineral leaching and root growth [16, 19, 20]. This *micro* scale environment generates a unique fluid behavior of the soil solution [20]: laminar hydrodynamic conditions dominate (Reynolds number <10), the surface-to-volume ratio increases noticeably with respect to systems of larger scales (*S*/*V* ∝ 1/L) and a readjustment of the surface charge occurs [21–23]. Thus far, no elements have been provided to identify the role of the microenvironment on mineral leaching.

Geochemistry literature [10, 14, 17, 18, 24–28] indicates that the leaching of K^+^ ions from K-feldspar follows two main stages in acidic solutions: i) a fast reaction, consisting of the exchange of surface K^+^ with H_3_O^+^ from the solution and ii) a slow reaction (10^−10^ to 10^-13^ mol_K+_ m^-2^ s^-1^), corresponding to the proton-catalyzed hydrolysis of Si-O and Al-O bonds in the framework structure. Over the long term (stage two), leaching rates show inverse dependence on time [25, 26]. However, little is known on the initial leaching period (stage one), i.e. the first instances of contact between mineral surfaces and soil solution, which are particularly important for the use of K-feldspar as a fertilizer. The surface reactivity in this early stage is unclear and could be due to loosely bound (defectual) exchangeable ions. However, other hypotheses have been brought forward, for example the absence of an altered layer at the mineral surface, which would form only in the later stages of weathering [14, 18, 28].

Such considerations call for new approaches to investigate soil minerals, within a space scale representative of the rhizosphere and within a time scale representative of agronomic rather than geological cycles. Therefore, with the aim to elucidate i) the effect of the soil microfluidic network on mineral leaching and ii) the potential agronomic benefit of K^+^ released from K-feldspar, we constructed a microfluidic device where leaching of an ultrapotassic syenite (S1 Materials and Methods) takes place in a microfluidic environment. Experimental results obtained with this device demonstrate that affordable silicate minerals can be exploited as a source of crop nutrients, provided that their application to agricultural fields is engineered by taking into account the microfluidic nature of soils.

## Materials and Methods

**Fig 1:**
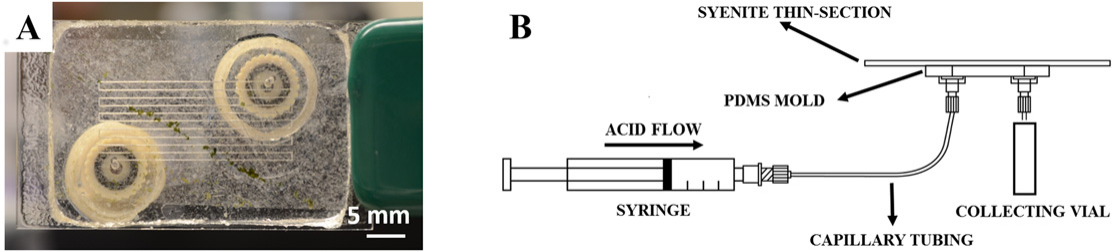
Microfluidic device to investigate leaching of soil minerals. (A) Photograph of the microfluidic device used for measuring the K^+^ leaching rate from the surface of an ultrapotassic syenite (K-feldspar>90 wt %). The serpentine channel (*~*30 cm long) is visible (see S1 Video for a visualization of the flow pattern). (B) Schematic of the microfluidic setup. The syringe is loaded with the leaching solution (HNO_3_ 1M), and connected to the inlet of the device by capillary tubing. The outlet of the device drains solution in a collecting vial and its K^+^ content is analyzed by Inductively Coupled Plasma Mass Spectroscopy (ICP-MS). The collecting vial is used to accumulate approximately 300 *μ*L and is continuously replaced.

The microfluidic device for the investigation of minerals leaching is assembled by sealing a thin section of the syenite sample to a polydimethylsiloxane (PDMS) mold, previously ablated by a laser to create a serpentine microchannel. A photograph and a schematic of the device are given in Fig 1(A) and Fig 1(B), respectively. Details of the experimental setup are provided below.

### Petrographic thin section

Petrographic thin sections (Spectrum Petrographics Inc.) of the syenite sample (S1 Materials and Methods) were 27 mm x 46 mm, two-sided polish (0.5 μm diamond), 30 μm thick and mounted on borosilicate glass with acrylic resin. Thin sections were fabricated using a synthetic kerosene (IsoparTM L (AM), ExxonMobil) to avoid contact with water. Three microfluidic kinetic experiments were conducted, each corresponding to a different thin section device, obtained from the same rock sample. Petrographic observation showed that the spatial distribution of the mineral grains among the three thin sections was very similar.

### Microchannel

Polydimethylsiloxane (PDMS) was obtained by mixing the elastomer base with the curing agent (SYLGARD® 184 silicone elastomer kit, Dow Corning) in a 1:10 weight ratio. The mixture was degassed under vacuum to remove trapped bubbles of air and cast on a custom made aluminum circular mold that hosted a silicon wafer (Sigma Aldrich) at the bottom. After casting, the mold was placed in a laboratory oven at 100°C for 45 minutes, to heat cure the degassed polymer mixture. The heat cured PDMS was removed from the mold and a microchannel ablated with a CO_2_ laser (Universal Laser System, V460, 60W) operated with the software CorelDRAW X419. Printing settings were as follow: vector pen mode, power 10%, speed 10%, PPI 1,000. Three PDMS molds were created, each corresponding to a specific microchannel, a specific thin section and a specific flow experiment. The cross section of each channel was triangular (S3 Fig). A summary of the microchannels’ dimensions measured by optical microscopy is given in S1 Table.

### Device Assembly and Microfluidic Flow Setup

A puncher (Harris Unicore 1.20) was used to pierce the PDMS at the extremities of the microchannel, to create the inlet and outlet. The PDMS was then washed by multiple acid/rinse cycles in standardized HNO_3_ 1M (Alfa Aesar) and water, respectively. The water used for washing as well as for the preparation of all solutions and dilutions was purchased from Ricca Chemical Company^®^ (ACS reagent grade, ASTM Type I, ASTM Type II).

The microfluidic device was constructed by creating a watertight seal between the thin section and the washed PDMS mold, which were both exposed to plasma cleaning (Harrick Scientific PDC 32G) in an oxygen atmosphere (67 Pa) for 55 s, and quickly contacted against each other immediately after the treatment [29, 30]. Due to natural variations within a rock sample, the microchannel was aligned each time over a different distribution of mineral grains, although for each flow experiment K-feldspar was by far the main mineral exposed to the leaching solution (thin sections were 94.5 wt % K-feldspar as discussed in S1 Materials and Methods).

To allow liquid in and out of the microfluidic device, Nanoports^TM^ (Idex Health&Science, N333) were used to connect capillary tubing (Idex Health&Science, 4010) to the microchannel. The capillary tubing at the inlet (6 cm long, I.D. 254 μm) was connected to a syringe (Becton, Dickinson and Company, plastics 10 mL) through a female Luer to microtight assy (Idex Health&Science, P 662). All capillary tubing and connections were washed by multiple acid/rinse cycles with standardized HNO_3_ 1M (Alfa Aesar) and water, respectively. The syringe (also pre-washed) was loaded with standardized HNO_3_ 1M (Alfa Aesar), and positioned on a pump (Harvard apparatus, PHD Ultra 703009) set to dispense the acid in the microchannel at the chosen flow rate *F* (S1 Table). The capillary tubing at the outlet (8 cm long, I.D. 254 μm) served as a drain for collection of the solution in propylene vials (Corning, 430289). The collecting vial was changed at regular time intervals depending on the flow rate, in order to collect about 300 μL at a time.

The inlet and outlet capillary tubing had a total volume of approximately 7.1×10^−9^ m^3^, which corresponded to residence times of ~2 min to ~26 s for the flow rate range of investigation. Such dead times and volumes, comparatively small with respect to the sampling time and sampling volume, did not affect our results. A schematic of the overall experimental setup is given in Fig 1B.

**Fig 2:**
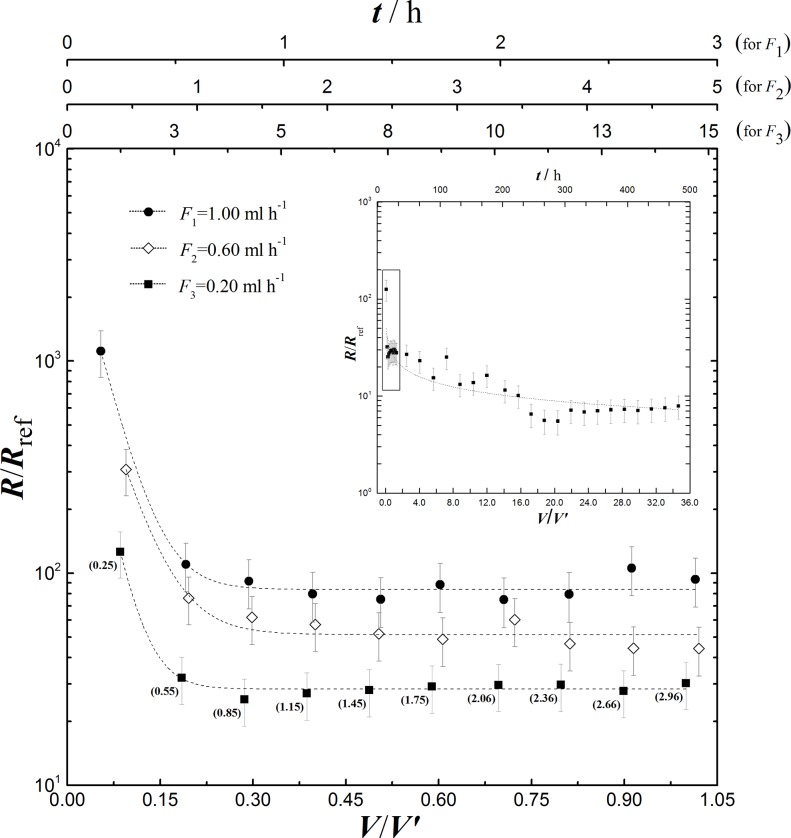
Microfluidic leaching rates of K^+^ ions (HNO_3_ 1M). Three dataset are reported, each corresponding to a specific flow experiment (S1 Materials and Methods, S1 Table): i) *F*_1_ = 1.0 mL h^-1^; *Re* = 2.0; *S*/*V* ~1.1×10^4^ m^-1^ ii) *F*_2_ = 0.6 mL h^-1^; *Re* = 0.9; *S*/*V* ~8.4×10^3^ m^-1^ iii) *F*_3_ = 0.2 mL h^-1^; *Re* = 0.3; *S*/*V* ~1.1×10^4^ m^-1^. *Re* is the Reynolds number. *R* is the experimental rate obtained from K^+^ concentrations determined by ICP-MS (S1 Materials and Methods); *R*_ref_ = 1.8×10^-10^ mol m^-2^ s^-1^ [18]. *V* is the cumulative volume of leaching solution in each experiment, shown as a bold number in parenthesis only for *F*_3_; *V’* = 2.96 mL (total volume accumulated at *F*_3_). In the top *x*-axes *t* is the cumulative time of leaching experiments. The inset shows the leaching rate over ~500 hours at *F*_3_ (the black box highlights the short-term leaching, which is showed in the main part of the graph). Determination of error bars is given in S3 Text. Curves through experimental points are provided exclusively to guide the eye.

The samples collected at the outlet were carefully weighted, diluted immediately in 0.5 g of water, capped and analyzed within 24 hours by Inductively Coupled Plasma Mass Spectroscopy (ICP-MS), as detailed in the S1 Materials and Methods and S2 Table. An exception was the long leaching experiment (see inset of Fig 2), where a number of samples was pooled without diluting, and analyzed all at once. In this case, samples were kept in a laboratory fridge (4°C) until analysis (two weeks maximum). In all experiments, the weight of the sample was used to back calculate the flow rate at the outlet (specific gravity of the leaching solution assumed to be 1 g cm^-3^), which was always found to be within 99.999% the input flow rate (S4 Fig). All experimental procedures were carried out at room temperature (19±2°C).

### Removal of the PDMS mold after microfluidic leaching

At the end of the microfluidic kinetic experiment, the PDMS was detached mechanically from the thin section with the aid of a spatula or a blade (North American 55411–050, Surgical carbon steel). The detached PDMS was cross-sectioned in at least three parts, to evaluate the channel depth (*h*) at different channel locations (S1 Table).

The surface of the syenite was slightly damped with a cloth moisten with ethanol (Sigma-Aldrich, ≥99.5%), to remove the larger PDMS debris. A very thin-layer of PDMS remained attached to the syenite surface allowing the determination by optical microscopy of the surface area (*S*) exposed to leaching (S5 Fig). Image analysis of the optical photographs was conducted by using a combination of the software ImageJ (http://imagej.nih.gov/ij/) and Adobe Photoshop^®^. The length of the “short arm” of the serpentine (S5A Fig) was set to 1 mm, according to the specification given to the channel in CorelDraw^®^ at the time of ablation, and verified independently by measurements with the optical microscope. Once the scale was set, the channel width was measured in different parts of the serpentine and an average was taken. At the inlet and outlet, the channel was round (S5C Fig) so that an average diameter was calculated. Then, the total surface area used to compute the leaching rate (S3 Text) was obtained by adding together all the contributing areas determined via analysis of the photographs. Further to the observations with the optical microscope, mineral surfaces were analyzed with a Scanning Electron Microscope (SEM), as detailed in the S1 Materials and Methods.

## Results and Discussion

The time-integrated leaching rate of K^+^ ions (S3 Text) measured at the outlet of the microfluidic device (*R*), is divided by a reference value (*R*_ref_) obtained from extrapolation of literature data reported in batch experiments [18]. Microfluidic-leaching experiments were conducted with 1 M HNO_3_, to ensure K^+^ concentration analyses with acceptable sensitivity within the experimental constraints (S1 Materials and Methods). In natural settings humic and small organic acids exuded by roots accelerate the rate of K^+^ leaching, and operating at high acidity allowed us to approximate such conditions. Choosing such an acidic leaching solution permits demonstrating with precision how microfluidic flow affects the variation of leaching rates with time, the primary objective of this work.

As shown in Fig 2, for all flow rates the microfluidic leaching rates in the apparent steady state are higher than expected, and enhancement more pronounced at the highest flow rate. Although the influence of micro-confinement effects cannot be ruled out for the data shown in Fig 2, such an enhanced K^+^ release can be presumed to originate from the short exposure time of the mineral surface to the leaching solution (15 hours for the slowest flow rate *F*_3_). After 500 hours, *R*/*R*_ref_ = 8±2 at 0.2 mL h^-1^ (inset in Fig 2) confirming this interpretation: in such microfluidic experiments, K^+^ leaching rates from syenite surfaces have not yet reached a true steady state but are actually transitioning from ion exchange to framework dissolution. The apparent “plateau” observed in Fig 2, together with *R*/*R*_ref_ values as high as ~10^3^ at short contact times (12 minutes), are important features of microfluidic leaching, which were not previously resolvable. Here, we demonstrate that by modulating the flow conditions, the release of K^+^ ions can be tuned, and sustained at an unexpectedly high value for an extended period of time. Such tuning occurs naturally in agricultural soils, where mineral surfaces are exposed to seasonal changes of flow conditions, for example due to rainfall. Therefore, at the spatial and temporal resolution experienced by roots in soil microenvironments K^+^ becomes available at a higher rate than previously reported. Results reported in Fig 2 shed new light on the processes by which plants can assimilate nutrients from geological materials in soil environments, and a microfluidic device enables controlled investigations of the underlying mechanisms.

The rate enhancement reported in Fig 2 suggests the opportunity to reconsider stonemeal, particularly in those countries that cannot afford or cannot access traditional potassium fertilizers [13–15]. In S1 Text we show that if microfluidic leaching rates are achieved in soils, reasonable amounts of syenites could provide sufficient potassium to sustain crop demand.

**Fig 3:**
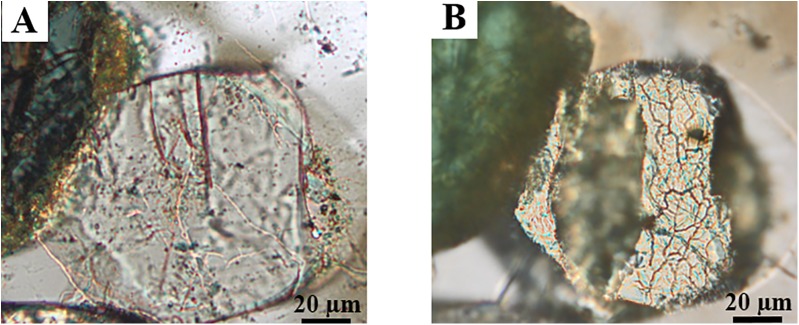
Optical photographs (cross-polarized light) of the bare surface of accessory apatite included in a syenite thin section. In photograph (**A**) the grain of apatite is in the thin section as received, before sealing the PDMS mold at the surface. In photograph (**B**) the grain of apatite has been exposed to microfluidic leaching at 0.2 mL h^-1^ (HNO_3_ 1 M). The entirety of the grain surface is centered with the microchannel (S6 and S7 Figs). These images are obtained from an independent thin section experiment and do not need to be related to the kinetic curves reported in Fig 2.

Furthermore, our approach is not limited to potassium minerals and is relevant for the investigation of soil kinetics of other nutrients and pollutants. For example, the true dynamics of phosphorous in soil microenvironments as well as its interactions with other chemical species are not known. Our device and method demonstrate both qualitatively and semi-quantitatively the dissolution of a grain of apatite, Ca_5_(PO_4_)_3_(F,OH), which is included in the syenite thin section as a trace mineral (Fig 3; S6 and S7 Figs). The bulk dissolution rate of igneous fluoroapatite is in the order of ~10^-6^ mol m^-2^ s^-1^ at pH = 0, significantly faster than the dissolution of K-feldspar [31]. Therefore, an optical microscope suffices to individuate local areas of preferential dissolution (Fig 3B), an observation that could be done in live mode and completed with post-leaching elemental analysis by energy dispersive spectroscopy (S1 Materials and Methods; S7 Fig). The effect of microfluidic leaching on titanite, also present as an inclusion mineral in syenites, is shown in S8 Fig.

Soil processes encompass a wide range of length scales. Agriculture extends from vast field areas to the rhizosphere, which in turn extends from meter-sized roots to micron-sized hairs. We demonstrate that, at these smaller length scales, microfluidics is needed to advance our understanding of the dynamics of the soil solution. Precision agriculture and improvements of agronomic yields rely on such advancement. Here, we establish that stonemeal fertilizers should succeed if the relationship between microfluidic leaching, ion exchange and crop uptake is as we surmise. To date, ion exchange in soils can be described by several rate laws (e.g., zero or first order, parabolic diffusion equation), meaning that a single best equation cannot be selected to describe kinetic rates univocally [8, 17, 32]. Although a rigorous evaluation of leaching rates of framework elements (Si and Al) will be necessary to fully elucidate the microfluidic nature of K-feldspar leaching, our findings offer new considerations on flow conditions and leaching times that must be taken into account to redesign fertilization from geological sources.

## Supporting Information

S1 Fig Amount of syenite rock required for leeks fertilization. *m*_ha_ values obtained from Eq1 (S1 Text) using *R*_leek_ = 1.2×10^−9^ mol_K+_ s^-1^ per leek and SSA varying between 0.5 and 2.5 m^2^ g^-1^. *R* varies between 10^-7^ mol m^-2^ s^-1^ (fastest microfluidic rate determined in this study; see Fig 2 of the main text) and 10^-14^ mol m^-2^ s^-1^ (framework weathering rate obtained for batch tests at pH = 7). Cut-off lines are given at 10 t ha^-1^ (practical limit) and 1.737 t ha^-1^ (*m*_eq_; see S1 Text). Arrows show a variation of two orders of magnitude in *R*, corresponding to a variation of two orders of magnitude in *m*_ha_. The pH scale refers to leaching rates *R* determined in batch or flow-through apparatuses.
(TIF)

S2 Fig Microfluidic absorption of nutrients by crop roots. Schematic of nutrient uptake by roots in (A) *macro*fluidic system where flow, roots and soils are investigated by measuring average bulk values of the parameters of interest and (B) *micro*fluidic system where a thin layer of soil solution is isolated from the soil bulk and only the roots in that layer (black bold) are considered. *R* is the rate at which K^+^ ions are available at the root surface (*R*_2_>*R*_1_ as demonstrated by data in Fig 2 of the main text); *F* is the flow rate; [K^+^] is the concentration of potassium ions at the roots surface ([K^+^]_2_>[K^+^]_1_). Note that the two drawings (A) and (B) as well as the several parts of the leek are not on scale. The schematic is highly idealized. It depicts a leek growing in a soil in contact with a fresh surface of K-feldspar that leaches K^+^ ions upon contact with water. Water flows at an average flow rate *F*, in a parallel direction to that of the K-feldspar surface.
(TIF)

S3 Fig Example of microchannel cross section. The photograph refers to microchannel 2 (see S1 Table).
(TIF)

S4 Fig Microfluidic flow rates. Flow rates measured at the outlet of the microfluidic device for different input flow rates. Each flow rate correspond to a different microchannel (S1 Table): microchannel 1–1.0 mL h^-1^; microchannel 2–0.6 mL h^-1^; microchannel 3–0.2 mL h^-1^.
(TIF)

S5 Fig Optical photographs (reflected light) of the syenite thin section after PDMS removal. (A) “Short arm” and corner of the serpentine. (B) Middle part of the serpentine. (C) Inlet (Ø = 1.24 mm). In both (A) and (B) the average width of the channel is 233 μm. Photographs refer to microchannel 2 (S1 Table).
(TIF)

S6 Fig Optical photographs (reflected light) of a grain of apatite in a syenite thin section. (A) Grain in the thin section as received. (B) Grain exposed to microfluidic leaching at 0.2 mL h^-1^ (HNO_3_ 1 M) in a channel about 175 μm wide, and photographed after PDMS removal. The entirety of the grain surface is centered within the microchannel (Fig 3 of the main text). PYX = pyroxene; KFS = K-feldspar; AP = apatite; TI = titanite. Photographs are obtained from the same experiment of titanite (S7 Fig) and are not to be related to curves reported in Fig 2 of the main text.
(TIF)

S7 Fig EDS elemental mapping. Ca, P and Si mapping of some mineral grains in a syenite thin section before (top images) and after (bottom images) microfluidic leaching at 0.2 mL h^-1^ (HNO_3_ 1 M) in a channel about 175 μm wide. The dotted box highlights a grain of apatite centered within the microchannel (same grain as S6 Fig and Fig 3 of the main text). The red box highlights a grain of apatite not touched by the microchannel. Si mapping is shown for reference.
(TIF)

S8 Fig Optical photographs (cross-polarized light) of a grain of titanite in a syenite thin section. (A) Grain of titanite is in the thin section as received. (B) Grain exposed to microfluidic leaching at 0.2 mL h^-1^ (HNO_3_ 1 M) in a channel about 175 μm wide, and photographed after PDMS removal; the dotted box highlights the area with an obvious change in birefringence. The entirety of the grain surface is centered within the microchannel. KFS = K-feldspar; TI = titanite. Photographs are obtained from the same thin section experiment of apatite (above) and are not to be related to curves reported in Fig 2 of the main text.
(TIF)

S1 Materials and Methods (DOCX)

S1 Table Overview of microchannels used in the present study. (DOCX)

S2 Table Overview of ICP-MS analysis. (DOCX)

S1 Text (DOCX)

S2 Text (DOCX)

S3 Text (DOCX)

S1 Video Observation of the flow pattern in the microfluidic device. Video recorded from the top of the microfluidic device showing the flow pattern inside the serpentine microchannel. In this flow-visualization experiment, an aqueous solution of Panceu S (Macron Fine Chemicals^TM^) at a concentration of 1 g per 100 mL of water (Ricca Chemical Company^®^) is flowed inside the device at 0.6 mL h^-1^. Length of the serpentine is ~30 cm, width and depth of the channel are 166 μm and 222 μm, respectively. The Reynolds number Re is ~1.0.
(MOV)
