# Supplementary figures and images for: Microfluidic Leaching of Soil Minerals: Release of K+ from K Feldspar
Source: PLoS One. 2015 Oct 20;10(10):e0139979. doi: 10.1371/journal.pone.0139979 (PMC4613825; doi:10.1371/journal.pone.0139979)

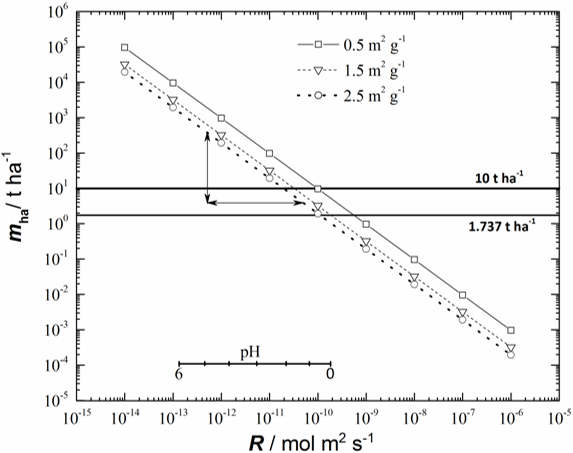

Supplement: S1 Fig — m ha values obtained from Eq1 (S1 Text) using R leek = 1.2×10^−9^ molK+ s-1 per leek and SSA varying between 0.5 and 2.5 m2 g-1. R varies between 10-7 mol m-2 s-1 (fastest microfluidic rate determined in this study; see Fig 2 of the main text) and 10-14 mol m-2 s-1 (framework weathering rate obtained for batch tests at pH = 7). Cut-off lines are given at 10 t ha-1 (practical limit) and 1.737 t ha-1 (m eq; see S1 Text). Arrows show a variation of two orders of magnitude in R, corresponding to a variation of two orders of magnitude in m ha. The pH scale refers to leaching rates R determined in batch or flow-through apparatuses. (TIF) [file pone.0139979.s001.tif]

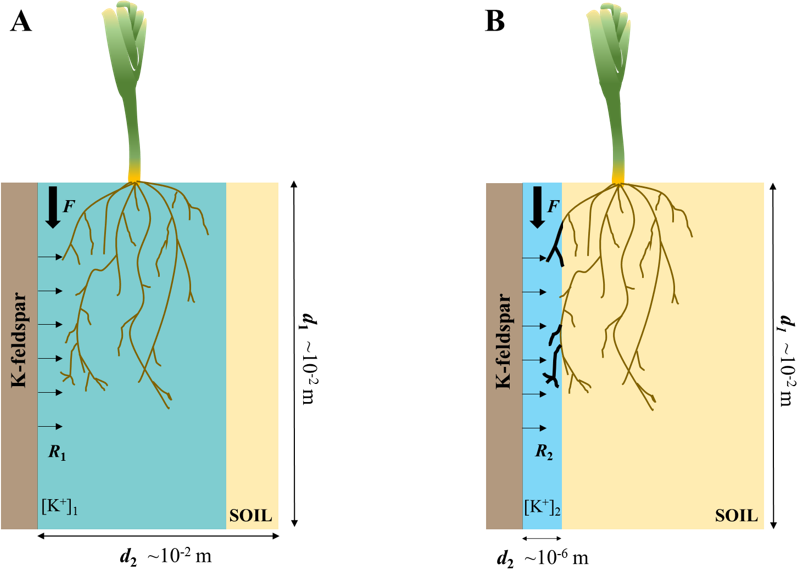

Supplement: S2 Fig — Schematic of nutrient uptake by roots in (A) macrofluidic system where flow, roots and soils are investigated by measuring average bulk values of the parameters of interest and (B) microfluidic system where a thin layer of soil solution is isolated from the soil bulk and only the roots in that layer (black bold) are considered. R is the rate at which K+ ions are available at the root surface (R 2>R 1 as demonstrated by data in Fig 2 of the main text); F is the flow rate; [K+] is the concentration of potassium ions at the roots surface ([K+]2>[K+]1). Note that the two drawings (A) and (B) as well as the several parts of the leek are not on scale. The schematic is highly idealized. It depicts a leek growing in a soil in contact with a fresh surface of K-feldspar that leaches K+ ions upon contact with water. Water flows at an average flow rate F, in a parallel direction to that of the K-feldspar surface. (TIF) [file pone.0139979.s002.tif]

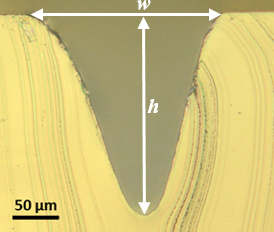

Supplement: S3 Fig — The photograph refers to microchannel 2 (see S1 Table). (TIF) [file pone.0139979.s003.tif]

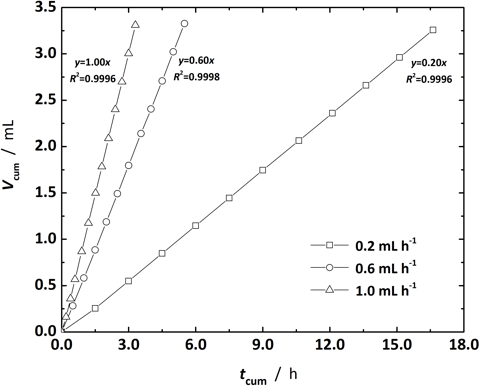

Supplement: S4 Fig — Flow rates measured at the outlet of the microfluidic device for different input flow rates. Each flow rate correspond to a different microchannel (S1 Table): microchannel 1–1.0 mL h-1; microchannel 2–0.6 mL h-1; microchannel 3–0.2 mL h-1. (TIF) [file pone.0139979.s004.tif]

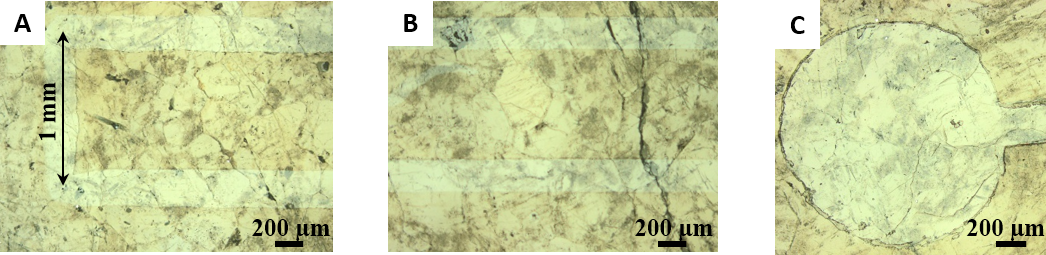

Supplement: S5 Fig — (A) “Short arm” and corner of the serpentine. (B) Middle part of the serpentine. (C) Inlet (Ø = 1.24 mm). In both (A) and (B) the average width of the channel is 233 μm. Photographs refer to microchannel 2 (S1 Table). (TIF) [file pone.0139979.s005.tif]

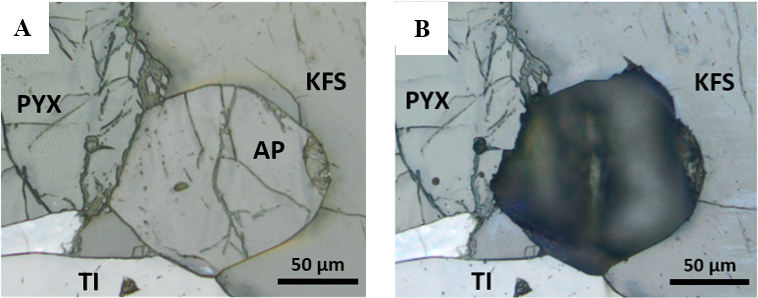

Supplement: S6 Fig — (A) Grain in the thin section as received. (B) Grain exposed to microfluidic leaching at 0.2 mL h-1 (HNO3 1 M) in a channel about 175 μm wide, and photographed after PDMS removal. The entirety of the grain surface is centered within the microchannel (Fig 3 of the main text). PYX = pyroxene; KFS = K-feldspar; AP = apatite; TI = titanite. Photographs are obtained from the same experiment of titanite (S7 Fig) and are not to be related to curves reported in Fig 2 of the main text. (TIF) [file pone.0139979.s006.tif]

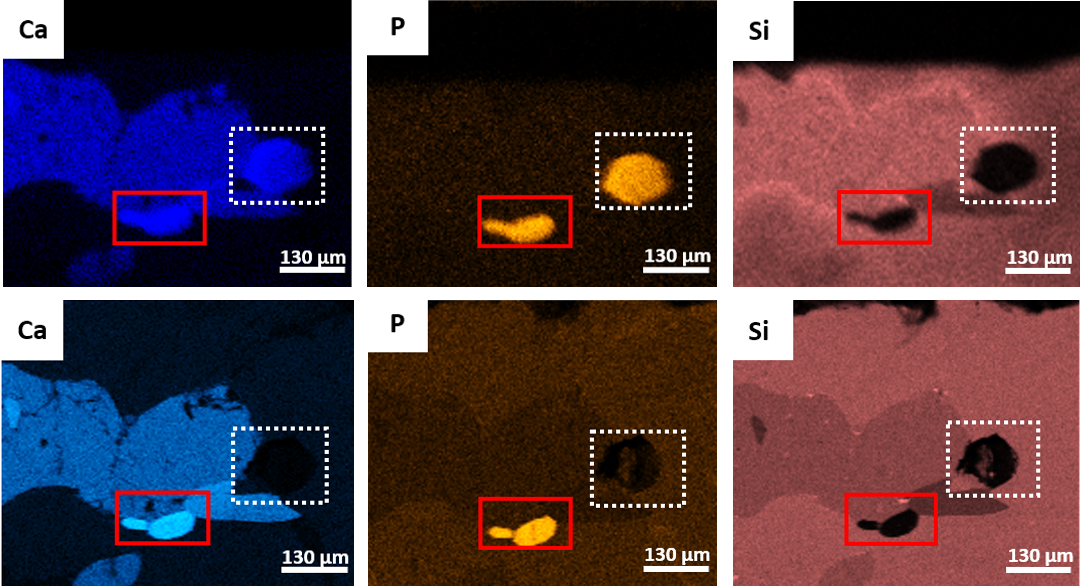

Supplement: S7 Fig — Ca, P and Si mapping of some mineral grains in a syenite thin section before (top images) and after (bottom images) microfluidic leaching at 0.2 mL h-1 (HNO3 1 M) in a channel about 175 μm wide. The dotted box highlights a grain of apatite centered within the microchannel (same grain as S6 Fig and Fig 3 of the main text). The red box highlights a grain of apatite not touched by the microchannel. Si mapping is shown for reference. (TIF) [file pone.0139979.s007.tif]

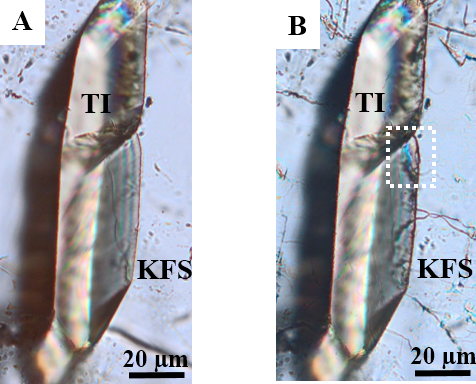

Supplement: S8 Fig — (A) Grain of titanite is in the thin section as received. (B) Grain exposed to microfluidic leaching at 0.2 mL h-1 (HNO3 1 M) in a channel about 175 μm wide, and photographed after PDMS removal; the dotted box highlights the area with an obvious change in birefringence. The entirety of the grain surface is centered within the microchannel. KFS = K-feldspar; TI = titanite. Photographs are obtained from the same thin section experiment of apatite (above) and are not to be related to curves reported in Fig 2 of the main text. (TIF) [file pone.0139979.s008.tif]
