# Supplementary material for: Microfluidic Leaching of Soil Minerals: Release of K+ from K Feldspar
Source: PLoS One. 2015 Oct 20;10(10):e0139979. doi: 10.1371/journal.pone.0139979 (PMC4613825; doi:10.1371/journal.pone.0139979)
Supplement: S1 Materials and Methods — (DOCX) [file pone.0139979.s009.docx]

Supporting Materials and Methods

**SYENITE SAMPLE**

The syenite sample (a rock specimen ~500 g) was obtained from the Triunfo batholith located between the municipalities of Triunfo and Serra Talhada, Pernambuco State, Brazil. The bedrock of the batholith is more than 200 m deep whereas elevation above sea level varies between ~460 m and ~830 m. The batholith (38’12”W, 7’55”S) is located about 400 km from the Atlantic Ocean, in the transversal zone of the Borborema province, which is a tectonic location characterized by granitic magmatism and extensive shear zones associated with the Neoproterozoic Brazilian orogeny (800-500 Ma).

The Triunfo batholith is composed of alkali feldspar syenites with inclusions of alkali pyroxenite. Syn plutonic and late-stage dikes are also present, which mineral phases are similar to those of the syenite but in different proportions. Local cataclastic transformations are observed, mainly progressive fracturing of existing rocks associated with fault zones.

The region is part of the Caatinga biome. The soils surrounding the site are Oxic Haplustept, Typic Haplustept and Typic Haplustult. The rainy period is limited to ~4 months a year, with total average rainfalls of 600 mm to 800 mm per year.

The mineralogical composition of the syenite was obtained by Rietveld refinement (not shown) of the X-rays diffraction (XRD) pattern of a ground sample. K feldspar (KAlSi_3_O_8_) was 94.5 wt % with pyroxene (3.9 wt %) and albite (1.5 wt %) as inclusion. Some grains of apatite (~50 150 µm), titanite and barite could also be observed in the thin section. The elemental analysis (not shown) performed with X‑rays fluorescence (XRF) revealed a K_2_O content of 14.3 wt %, classifying the syenite as ultrapotassic. The K_2_O content measured by XRD was within 12% of that one measured by XRF.

**LEACHATE SOLUTION ANALYSIS BY INDUCTIVELY COUPLED PLASMA MASS SPECTROSCOPY (ICP-MS)**

ICP‑MS (Agilent Technologies 7700 Series) was used to determine the concentration of potassium collected at the outlet of the microfluidic device. The calibration curve was built from fresh volumetric standards (Table S2), prepared by appropriate dilutions of a solution at 1,000 ppm of K (TraceCERT^®^, Fluka Analytical) in 0.5 M HNO_3_, which was freshly prepared by dilution of standardized HNO_3_ 1M (Alfa Aesar). ^39^K (isotopic abundance 93.26%) was chosen as the analyte. The instrument used an Octopole Reaction System (ORS), which was run in He mode (He=4.0 mL min^-1^) [1]. When appropriate, an acidic solution (HNO_3_, pH=2) of In at 1 ppm was used as Internal Standard (IS) (Table S2). To minimize analytical error, three wash cycles at increasing concentration of HNO_3_ (10^‑2^M, 10^‑1^ M and 0.5 M) were performed between samples, which were analyzed from the most diluted to the most concentrated. The stability graph of the internal standard was always within the set boundaries of ± 20% recovery. Each sample was analyzed in three replicates and a Relative Standard Deviation (RSD) ≤5% was obtained for all samples. The Background Equivalent Concentration (BEC) [2] was chosen as a parameter to reject or retain concentration readings (Table S2). All concentration values processed to obtain microfluidic leaching rates were above the BEC value.

A possible source of systematic error were K^+^ impurities in the HNO_3_ 1 M purchased from Alfa Aesar, and used as the leachant for all microfluidic experiments. We performed a series of independent tests (not shown) to determine if such impurities were effectively present. For this set of tests, the standardized HNO_3_ 1 M from Alfa Aesar was the sample analyzed by ICP‑MS, whereas ultrapure water (Tamapure‑AA, K^+^<10 ppt, Tama Chemicals CO., LTD), ultrapure HNO_3_ (Tamapure‑AA‑10, K^+^<10 ppt, Tama Chemicals CO., LTD) and a solution at 1,000 ppm of K (TraceCERT^®^, Fluka Analytical) were used to build the calibration curve, which was obtained from a quadratic fitting. For this specific analysis, data were below the BEC value. However, the RSD for three replicates of the same sample was ≤ 5% and the RSD for five different samples determination was ≤ 5%. Therefore, the obtained experimental concentrations were not rejected and the K^+^ impurity content was determined to be 8.5×10^‑7^ ± 4×10^‑8^ M. An overview of the ICP‑MS analysis, including Detection Limits (DL) are given in Table S2.

**OPTICAL MICROSCOPE**

An optical microscope (Olympus, BX51) equipped with a digital camera (Olympus UC31) was used to photograph the thin‑section before and after exposure to acid in the microfluidic device. Olympus Stream (Essentials license) [3] was used as the software to elaborate the photographs. Exposure settings and rotation angle of the cross‑polarizer varied, depending on the thin‑section and mineral being photographed. An example of a grain of apatite observed in transmitted light is given in the main text (Fig.3). The same grain observed in reflected light before and after microfluidic leaching is given in Fig.S6. The original composition of the apatite grain as obtained by point mapping at the center of the grain with Energy Dispersive X‑ray Spectroscopy was O=50.9 wt %, F=5.9 wt %, P=19.2 wt % and Ca=24.1 wt %. After leaching, the composition at the same location was O=67.8 wt %, Al=7.9 wt %, Si=16.1 wt%, Cl=3.0 wt %, P=1.0 wt % and Fe 2.3 wt %. This analysis suggests a possible re‑precipitation of new phases or the emerging of Fe‑minerals at the bottom of the dissolved apatite (Fig.S7).

Titanite (CaTiSiO_5_) is also among the inclusion minerals present in the syenite used in this study. A photograph of a grain of titanite before and after microfluidic leaching is given in Fig.S8. It is shown that microfluidic leaching changes the birefringence of the mineral grain when observed in transmitted cross‑polarized light. It is an indication of modifications in the atomic arrangements constituting the mineral structure caused by the leaching. Fig.S8 demonstrates that microfluidic devices like the one presented here, could potentially expand the current usage of petrographic thin‑section, which do not always allow the identification of mineral phases by simple observations with an optical microscope. Here, we show that depending on intrinsic dissolution rates, more mineral distinctions become possible.

**SCANNING ELECTRON MICROSCOPE (SEM)**

The Scanning Electron Microscope (JEOL 6610 LV) was equipped with an Energy Dispersive X‑ray Spectroscopy (EDS) system, with a silicon drift detector. It was used to obtain maps of the elemental composition of different mineral grains included in the feldspar. The thin-sections were not gold coated and, since the syenite is not conductive, exhibited strong charging. Although this made difficult individuating the grains of interest, it did not affect the EDS signal, which was performed in a high vacuum chamber at a working distance from the detector of 15 mm. The software used for elemental mapping was Iridium Ultra^TM^. The mapping of the apatite grain shown both in Fig.3 of the main text and Fig.S6, is given in Fig.S8. Both Ca and P leached only from the grain effectively centered within the microchannel.

References

1. http://www.chem.agilent.com/Library/articlereprints/Public/5990_7574EN.pdf

2. V. Thomsen, Spectral Background Radiation and the Background Equivalent Concentration in Elemental Spectrochemistry. *Spectroscopy* **27**, 28-36 (2012).

3. http://www.olympus-ims.com/en/microscope/stream/
