# Supplementary material for: Microfluidic Leaching of Soil Minerals: Release of K+ from K Feldspar
Source: PLoS One. 2015 Oct 20;10(10):e0139979. doi: 10.1371/journal.pone.0139979 (PMC4613825; doi:10.1371/journal.pone.0139979)
Supplement: S1 Table — (DOCX) [file pone.0139979.s010.docx]

Supporting Table S1

Table S1. Overview of microchannels used in the present study^‡^.

|  | ***F*** (mL h^-1^) |  | | Measurements | | | | | |
| --- | --- | --- | --- | --- | --- | --- | --- | --- | --- |
|  |  | ***w*** (µm) | ***h*** (µm) | | ***A*** (m^2^) | ***V”*** (m^3^) | ***L*** (m) | ***t”*** (s) | ***S*** (m^2^) |
| Microchannel 1 | 1.0 | 159 | 182 | | 1.5×10^-8^ | 4.0×10^-9^ | 0.28 | ~15 | 4.3×10^-5^ |
| Microchannel 2 | 0.6 | 233 | 226 | | 2.6×10^-8^ | 7.3×10^-9^ | 0.28 | ~44 | 6.2×10^-5^ |
| Microchannel 3 | 0.2 | 310 | 159 | | 2.5×10^-8^ | 6.9×10^-9^ | 0.28 | ~120 | 7.3×10^-5^ |

^‡^ Width (*w*) and height (*h*) are given as the average of at least 5 measurements made by observation with an optical microscope (BX51, Olympus) in reflected light, at different location of the channel. *A* is the cross‑sectional area and *V”* the volume of the channel, respectively. *t”* is the residence time of the leaching solution in the microchannel. The surface area (*S*) is determined according to the procedure described in Materials and Methods.
