# Supplementary material for: Microfluidic Leaching of Soil Minerals: Release of K+ from K Feldspar
Source: PLoS One. 2015 Oct 20;10(10):e0139979. doi: 10.1371/journal.pone.0139979 (PMC4613825; doi:10.1371/journal.pone.0139979)
Supplement: S2 Table — (DOCX) [file pone.0139979.s011.docx]

Supporting Table S2

Table S2. Overview of ICP‑MS analysis.

|  | Standards (ppb K^+^) | Quality control parameters | | |
| --- | --- | --- | --- | --- |
|  |  | **BEC** (M) | **DL** (M) | **IS** (-) |
| Microchannel 1 | 0, 25, 50, 150, 300, 450, 600 | 1.9×10^‑6^ | 6×10^‑8^ | Yes |
| Microchannel 1a^⸿^ | 0, 25, 50, 150, 300 | 1.3×10^‑6^ | 1×10^‑7^ | Yes |
| Microchannel 1b^⸿^ | 0, 25, 50, 150, 300 | 9.5×10^‑7^ | 3×10^‑8^ | Yes |
|  |  |  |  |  |
| Microchannel 2 | 0, 50, 150, 300, 450 | 9.2×10^‑7^ | 6×10^‑8^ | Yes |
|  |  |  |  |  |
| Microchannel 3 | 0, 50, 150, 300, 450 | 1.6×10^‑6^ | 5×10^‑8^ | No |
|  |  |  |  |  |
| HNO_3_ 1M | 0, 20, 40, 60, 80 | 1.6×10^‑6^ | 6×10^‑8^ | No |

^⸿^ Data are obtained from microchannel 1. “Microchannel 1a” and “Microchannel1b” are chosen as labels to indicate that two sets of ICP‑MS analysis were made to assess the K^+^ leaching rate in the long leaching experiment (data of the inset of Fig.2 of the main text).
