# Supplementary material for: Microfluidic Leaching of Soil Minerals: Release of K+ from K Feldspar
Source: PLoS One. 2015 Oct 20;10(10):e0139979. doi: 10.1371/journal.pone.0139979 (PMC4613825; doi:10.1371/journal.pone.0139979)
Supplement: S1 Text — (DOCX) [file pone.0139979.s012.docx]

Supporting Text 1 (S1 text)

**AGRONOMIC MOTIVATION OF THE PRESENT STUDY**

Potassium (K) is an essential crop nutrient together with nitrogen (N) and phosphorous (P). Potassium commodities are globally traded under the name of *potash*, with a total potassium content conventionally expressed as K_2_O wt % [1-8].

We estimate the amount of syenite per soil unit necessary to provide the amount of potassium needed by crops. We select leeks as a model crop due to their relatively short growth period (~150 days), ease of growth rate determination (growth is monitored by simply measuring the leek diameter) and detailed data available from a recent study [9]. Our approach can be adapted to other crops, especially those for which K‑fertilizer requirements are higher than leeks. However, such considerations are beyond the scope of this work. Tables of nutrients content in crops and food can be found in dedicated references [10, 11].

Our approach assumes that:

- The amount of potassium (K^+^) needed for leek growth is 230 kg ha^-1^ (equivalent to 0.59 mol_K+_ m^-2^ or 0.015 mol_K+_ per leek), as suggested in fertilization guidelines [9, 12]. This amount of potassium roughly correspond to the actual K content of leeks obtained by assuming an average mass of 200 g per leek, an average K content of 380 mg_K_/100 g_leek_ [10] and an average number of 380,000 leeks per hectare
- The soil depth accessed by leeks is 0.3 m per leek, the soil area accessed by leeks is 0.026 m^2^ per leek, corresponding to 38 leeks per 1 m^2^, and the soil volume accessed by the leek root system is 7.9×10^-3^ m^3^ per leek [9]
- The growth period is 150 days (1.3×10^7^ s)
- The K‑feldspar potassium equivalent (*m*_KFS_) needed by a leek is 4.32 g, corresponding to a syenite potassium equivalent (*m*_eq_) of 4.57 g (assuming the K‑feldspar content of the syenite to be 94.5 wt %)
- Effects of temperature, irrigation, quality of soil, nutrients supplies (other than K), biological activity, etcetera, are not considered

We proceed as follow:

- We calculate the rate of K^+^ uptake per leek under the set of assumptions listed above. This is equivalent to *R*_leek_=1.2×10^‑9^ mol_K+_ s^-1^ per leek
- We calculate the mass of syenite per hectare *m*_ha_ (t ha^-1^) which, for any given leaching rate and Specific Surface Area (SSA), provides a total surface that suffices to leach the amount of K^+^ needed by a single leek, within its growth period. The SSA of ground syenites is assumed to vary between 0.5 and 2.5 m^2^ g^-1^. The K^+^ leaching rate from the syenite surface (*R*) varies between a) the fastest *ion-exchange* microfluidic rate determined in this study for *F*_1_=1 mL h^‑1^ (i.e. 10^‑7^ mol m^-2^ s^‑1^; Fig.2 of the main text) and b) the *framework weathering* rate obtained for batch tests at pH=7 (10^‑14^ mol m^‑2^ s^‑1^) [13].

Eq.1 is used to compute *m*_ha_ values:

$m_{\mathrm{ha}}=\frac{{n\times R}_{\mathrm{leek}}}{SSA\times R}$ Eq.1

where *n*=380,000 since we focus on a hectare of crop cultivation. Eq.1 is plotted in Fig.S1.

Our approach implies a constant syenite surface not consumed over time, and that leaches continuously K^+^ ions during the growth period, at a constant rate. Therefore, Eq.1 does not take into account the need for *m*_eq_. In Fig.S1, a lower bound and upper bound cut‑off values are shown, at *m*_eq_ and 10 t ha^-1^, respectively. The upper bound is determined by considering a mass of syenite comparable to that of gypsum, which is commonly used to amend agricultural soils [14, 15].

For all combinations below the lower bound cut‑off, the syenite would be consumed before the growth period and multiple applications (not determined in this study) would be needed. The shrinking core model can be used to refine this approach [16]. The model can predict the time at which particles of ground syenites will have completely leached out all the available K. Such a refinement requires knowledge of mass transfer coefficients in soil environments, which are generally unknown or difficult to determine experimentally.

For all combination between the cut‑off planes, the application of ground syenites in soils may benefit agriculture.

For all combinations above the upper bound cut‑off more syenite than that practically applicable to soils would be required.

We point out that for variations of two orders of magnitude in the leaching rate, the mass of syenite needed according to this mass‑balance (Eq.1) also changes by two orders of magnitude. Therefore, variations in leaching rates that occur during an agronomic cycle, for example due to irrigation, release of root exudates in different stages of crop growth or other environmental variables (e.g., rainfall and temperature) will affects substantially the forecast of the ground syenite needed as K‑fertilizer.

At neutral or near neutral pH the use of ground syenite (stonemeal [4, 5]) does not seem a feasible approach to potassium fertilization. However, for acidic soils such as those of tropical countries, K-feldspar and syenite rocks have the potential to be used as K‑fertilizer. If enhanced leaching due to ion exchange in microfluidic conditions holds true (Fig.2 of the main text), an even better scenario emerge, for which only minor amount of syenite (at minimum equal to *m*_eq_) would suffice to provide agronomic benefit.


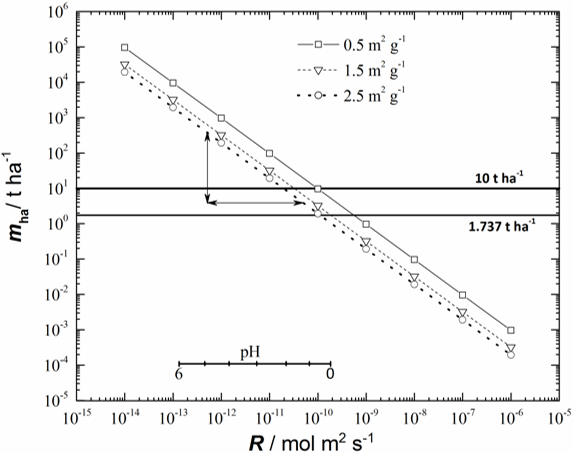


**Fig. S1. Amount of syenite rock required for leeks fertilization.** *m*_ha_ values obtained from Eq.1 (Supporting Text 1) using *R*_leek_=1.2×10^-9^ mol_K+_ s^-1^ per leek and SSA varying between 0.5 and 2.5 m^2^ g^‑1^. *R* varies between 10^‑7^ mol m^-2^ s^-1^ (fastest microfluidic rate determined in this study; see Fig.2 of the main text) and 10^‑14^ mol m^‑2^ s^‑1^ (framework weathering rate obtained for batch tests at pH=7). Cut-off lines are given at 10 t ha^-1^ (practical limit) and 1.737 t ha^-1^ (*m*_eq_; see Supporting Text 1). Arrows show a variation of two orders of magnitude in *R*, corresponding to a variation of two orders of magnitude in *m*_ha_. The pH scale refers to leaching rates *R* determined in batch or flow‑through apparatuses.

References

1. Ciceri D, Manning DA, Allanore A. Historical and technical developments of potassium resources. Science of The Total Environment. 2015;502:590-601.

2. Skorina T, Allanore A. Aqueous alteration of potassium-bearing aluminosilicate minerals: from mechanism to processing. Green Chemistry. 2015.

3. Manning DA. Mineral sources of potassium for plant nutrition. A review. Agronomy for Sustainable Development. 2010;30(2):281-94.

4. Leonardos O, Fyfe W, Kronberg B. The use of ground rocks in laterite systems: an improvement to the use of conventional soluble fertilizers? Chemical Geology. 1987;60(1):361-70.

5. Leonardos OH, Theodoro SH, Assad M. Remineralization for sustainable agriculture: A tropical perspective from a Brazilian viewpoint. Nutrient Cycling in Agroecosystems. 2000;56(1):3-9.

6. van Straaten P. Rocks for Crops: Agrominerals of sub-Saharan Africa. Nairobi, Kenya: ICRAF; 2002.

7. Römheld V, Kirkby EA. Research on potassium in agriculture: needs and prospects. Plant and Soil. 2010;335(1-2):155-80.

8. Henao J, Baanante CA. Estimating rates of nutrient depletion in soils of agricultural lands of Africa. Muscle Shoals, AL, USA: International Fertilizer Development Center 1999.

9. Mohammed S, Brandt K, Gray N, White M, Manning D. Comparison of silicate minerals as sources of potassium for plant nutrition in sandy soil. European Journal of Soil Science. 2014;65(5):653-62.

10. Finglas PM, Roe MA, Pinchen HM, Berry R, Church SM, Dodhia SK, et al. McCance and Widdowson's The Composition of Food. 7th ed. Cambridge: Royal Society of Chemistry2015.

11. United States Department of Agriculture. National Nutrient Database for Standard Reference Release 27 2015 [03/04/2015]. Available from: http://ndb.nal.usda.gov/ndb/nutrients/index.

12. DEFRA. Fertiliser Manual (RB209). 2010.

13. Blum AE, Stillings LL. Feldspar dissolution kinetics. In: Chemical Weathering Rates of Silicate Minerals. Reviews in Mineralogy and Geochemistry. 311995. p. 291-351.

14. Shainberg I, Sumner M, Miller W, Farina M, Pavan M, Fey M. Use of gypsum on soils: A review. Advances in Soil Science. 9. New York: Springer-Verlag; 1989.

15. Oster J. Gypsum usage in irrigated agriculture: a review. Fertilizer research. 1982;3(1):73-89.

16. Levenspiel O. Chemical reaction engineering. 2nd ed: John Wiley & Son, Inc.; 1972.
