# Supplementary material for: Microfluidic Leaching of Soil Minerals: Release of K+ from K Feldspar
Source: PLoS One. 2015 Oct 20;10(10):e0139979. doi: 10.1371/journal.pone.0139979 (PMC4613825; doi:10.1371/journal.pone.0139979)
Supplement: S2 Text — (DOCX) [file pone.0139979.s013.docx]

Supporting Text 2 (S2 text)

**LEACHING OF SOIL MINERALS AND ABSORPTION BY ROOTS**

Fig.S2 shows a schematic of the absorption by roots of K^+^ ions released by the surface of a mineral (e.g., K‑feldspar). At the macroscale (Fig.S2A) the rate at which potassium becomes available in the soil solution (*R*_1_) is commonly thought to be that one determined with conventional weathering apparatuses in geochemistry literature [1]. However, in a system with at least one dimension in the microscale domain (Fig.S2B), for example a root growing in a soil microchannel, the rate at which potassium is available (*R*_2_) is higher, as demonstrated by results shown in Fig.2 of the main text of this work. Furthermore, the K^+^ concentration in a soil pore is higher than the average measured for a bulk system. Microfluidic tools permit investigating leaching rate of mineral surfaces in microenvironments.

**
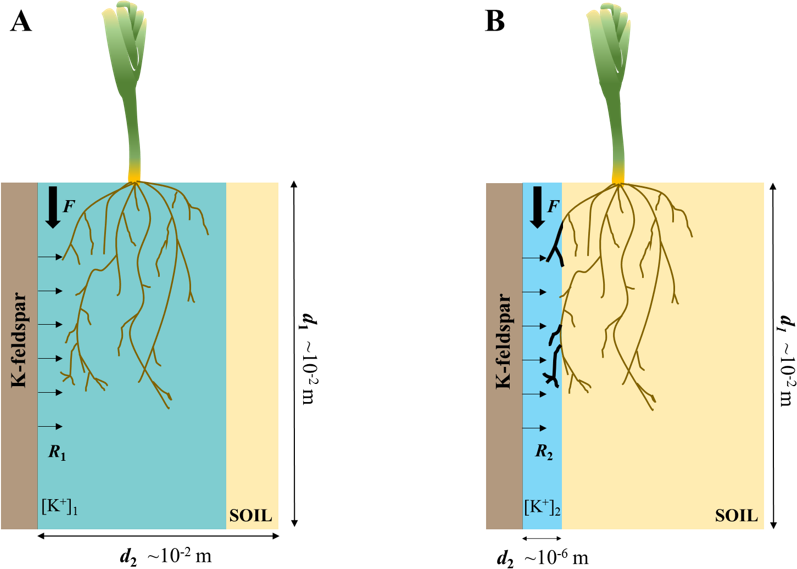
**

**Fig. S2 Microfluidic absorption of nutrients from roots.** Schematic of nutrient uptake by roots in (A) *macro*fluidic system where flow, roots and soils are investigated by measuring average bulk values of the parameters of interest and (B) *micro*fluidic system where a thin layer of soil solution is isolated from the soil bulk and only the roots in that layer (black bold) are considered. *R* is the rate at which K^+^ ions are available at the root surface (*R*_2_>*R*_1_ as demonstrated by data in Fig.2 of the main text); *F* is the flow rate; [K^+^] is the concentration of potassium ions at the roots surface ([K^+^]_2_>[K^+^]_1_). Note that the two drawings (A) and (B) as well as the several parts of the leek are not on scale. The schematic is highly idealized. It depicts a leek growing in a soil in contact with a fresh surface of K‑feldspar that leaches K^+^ ions upon contact with water. Water flows at an average flow rate *F*, in a parallel direction to that of the K‑feldspar surface.

References

1. Blum AE, Stillings LL. Feldspar dissolution kinetics. In: Chemical Weathering Rates of Silicate Minerals. Reviews in Mineralogy and Geochemistry. 311995. p. 291-351.
