# Supplementary material for: Microfluidic Leaching of Soil Minerals: Release of K+ from K Feldspar
Source: PLoS One. 2015 Oct 20;10(10):e0139979. doi: 10.1371/journal.pone.0139979 (PMC4613825; doi:10.1371/journal.pone.0139979)
Supplement: S3 Text — (DOCX) [file pone.0139979.s014.docx]

Supporting Text 3 (S3 text)

**K^+^ LEACHING RATE**

The kinetics of mineral leaching have been investigated extensively in both batch and flow‑through systems [1-11]. Different formalisms have been used to describe the rate, for example starting from thermodynamic data [3, 12] or more simply by making use of empirical measurements of concentrations and ion-fluxes [1, 4, 9]. Following textbooks of chemical engineering, rates can be determined from design equations of standard chemical reactor, for example the *plug-flow reactor*, which make use of macro‑scale bulk parameters such as mass transfer coefficients [4, 11, 13, 14]. The latest advances include computational simulations to model weathering within a framework of *reactive transport* in micro‑sized environments [3, 14]. This latter approach provides the most holistic interpretation of kinetics since it proposes a simultaneous mathematical treatment of both hydrodynamic and rate equations.

In the present study we calculate the leaching rate of K^+^ ions according to Eq.2, which is based on concentrations measured empirically [4, 9]:

$R=\frac{C_{\mathrm{out}}-C_{\mathrm{in}}}{\nu S}\frac{dV}{dt}$ Eq.2

where *R* (mol m^-2^ s^-1^) is the leaching rate of K^+^, *C*_out_ (mol L^-1^) and *C*_in_ (mol L^-1^) are the concentrations of K^+^ measured at the outlet of the device (collecting vial) and inlet (HNO_3_ leachant), respectively, ν (-) is the stoichiometric coefficient (equal to 1 for K in the feldspar), *S* (m^2^) is the surface area exposed to leaching, whereas *dV*/d*t* corresponds to the flow rate.

Eq.2 is rewritten in explicit form as:

$R=\frac{C_{\mathrm{out}}-C_{\mathrm{in}}}{S} F$ Eq.3

where *F* (L s^-1^) is the flow rate of the leaching solution as reported in Table S1. *C*_out_ was determined with ICP‑MS as discussed in Supplementary Methods and Materials; *C*_in_ was 8.5×10^‑7^±4×10^‑8^ M as discussed in Supplementary Methods and Materials; *S* was determined geometrically as discussed in Supplementary Methods and Materials.

The standard deviation on the experimental rate was computed from Eq.4:

$\sigma_{R}=R\sqrt{\frac{\sigma_{C_{\mathrm{out}}}^{2}+\sigma_{C_{\mathrm{in}}}^{2}}{\left( C_{\mathrm{out}}-C_{\mathrm{in}} \right)^{2}}+\frac{\sigma_{S}^{2}}{S^{2}}}$ Eq.4

which was obtained from error propagation theory [15], under the following set of assumptions:

The standard deviation on the concentration value at the outlet ($\sigma_{C_{\mathrm{out}}}$) was 10% the average value (*C*_out_) whereas the standard deviation on the inlet concentration (*C*_in_) was ± 4×10^‑8^ M (Supplementary Methods and Materials).

The standard deviation on the surface area value ($\sigma_{S}$) was 10% the area value (*S*); this is because despite a fair accuracy in the geometrical determination (Supplementary Methods and Materials) inclusion minerals were present on the channel paths (10 wt % of the bulk sample), reducing the feldspar area effectively exposed to leaching. The uncertainty on the flow rate was negligible, as shown in Fig.S4.

Finally, experimental data in Fig.2 of the main text are plotted as ratio of *R* to *R*_ref_, where *R*_ref_ is the value extrapolated from a survey of literature data obtained in batch experiments (i.e. 1.8×10^‑10^ mol m^-2^ s^-1^) [1]. This extrapolated value is in excellent agreement with the leaching rate value of 1.7×10^‑10^ mol m^‑2^ s^‑1^ obtained from an independent test (not shown) of a ground sample of the syenite in HNO_3_ 1M (syenite particles in the ground sample <150µm; specific surface area of the syenite particles according to the BET method 1.04 m^2^ g^-1^; 24 hours leaching; solid‑to‑liquid ratio=0.1).

Leaching rates obtained from different laboratories are in relatively poor agreement, within a factor of 5, owing to differences in mineral composition, microstructure, grain size, ionic strength, etcetera [1]. Furthermore, as discussed in the literature, for experiments conducted at human timescales a decrease in the rate value occurs over time [4]. Here, we associated to *R*_ref_ a standard deviation ($\sigma_{R_{\mathrm{ref}}}$) equal to 20% its value. Such choice is made assuming that the average $\sigma_{R}/R$ for our experiments applies also to literature data used for the extrapolation. The final error bars plotted in Fig.2 of the main text are obtained according to Eq.5:

$\sigma_{R/R_{\mathrm{ref}}}=\frac{R}{R_{0}}\sqrt{\frac{\sigma_{R}^{2}}{R^{2}}+\frac{\sigma_{R_{\mathrm{ref}}}^{2}}{R_{\mathrm{ref}}^{2}}}$ Eq.5

References

1. Blum AE, Stillings LL. Feldspar dissolution kinetics. In: Brantley AWaSL, editor. Chemical Weathering Rates of Silicate Minerals. Reviews in Mineralogy and Geochemistry. 311995. p. 291-351.

2. Wilson M. Weathering of the primary rock-forming minerals: processes, products and rates. Clay Minerals. 2004;39(3):233-66.

3. Zhu C. Geochemical modeling of reaction paths and geochemical reaction networks. Reviews in Mineralogy and Geochemistry. 2009;70(1):533-69.

4. White AF, Brantley SL. The effect of time on the weathering of silicate minerals: why do weathering rates differ in the laboratory and field? Chemical Geology. 2003;202(3):479-506.

5. AAVV. Reviews in Mineralogy. White AF, Brantley SL, editors. Washington D.C.: Mineralogical Society of America; 1995.

6. Amacher MC. Methods of obtaining and analyzing kinetic data. Rates of soil chemical processes: Soil Science Society of America; 1991. p. 19-59.

7. Van Hees P, Lundström U, Mörth C-M. Dissolution of microcline and labradorite in a forest O horizon extract: the effect of naturally occurring organic acids. Chemical Geology. 2002;189(3):199-211.

8. Lee MR, Hodson ME, Parsons I. The role of intragranular microtextures and microstructures in chemical and mechanical weathering: direct comparisons of experimentally and naturally weathered alkali feldspars. Geochimica et Cosmochimica Acta. 1998;62(16):2771-88.

9. Chou L, Wollast R. Study of the weathering of albite at room temperature and pressure with a fluidized bed reactor. Geochimica et Cosmochimica Acta. 1984;48(11):2205-17.

10. Schweda P, editor Kinetics of alkali feldspar dissolution at low temperature. 6th International Symposium Water/Rock Interaction; 1989; Rotterdam.

11. Bevan J, Savage D. The effect of organic acids on the dissolution of K-feldspar under conditions relevant to burial diagenesis. Mineralogical Magazine. 1989;53:415-25.

12. Lasaga AC, Soler JM, Ganor J, Burch TE, Nagy KL. Chemical weathering rate laws and global geochemical cycles. Geochimica et Cosmochimica Acta. 1994;58(10):2361-86.

13. Levenspiel O. Chemical reaction engineering. 2nd ed: John Wiley & Son, Inc.; 1972.

14. Li L, Steefel CI, Yang L. Scale dependence of mineral dissolution rates within single pores and fractures. Geochimica et Cosmochimica Acta. 2008;72(2):360-77.

15. Skoog D, West D, Holler F, Crouch S. Fundamentals of analytical chemistry. 9th ed: Brooks Cole/Cengage Learning; 2014.
